# The MADF-BESS Protein CP60 Is Recruited to Insulators via CP190 and Has Redundant Functions in *Drosophila*

**DOI:** 10.3390/ijms241915029

**Published:** 2023-10-09

**Authors:** Larisa Melnikova, Varvara Molodina, Valentin Babosha, Margarita Kostyuchenko, Pavel Georgiev, Anton Golovnin

**Affiliations:** 1 Department of Drosophila Molecular Genetics, Institute of Gene Biology, Russian Academy of Sciences, 34/5 Vavilov Street, Moscow 119334, Russia; lsm73@mail.ru (L.M.);; 2 Department of the Control of Genetic Processes, Institute of Gene Biology, Russian Academy of Sciences, 34/5 Vavilov Street, Moscow 119334, Russiageorgiev_p@mail.ru (P.G.); 3 Center for Precision Genome Editing and Genetic Technologies for Biomedicine, Institute of Gene Biology, Russian Academy of Sciences, 34/5 Vavilov Street, Moscow 119334, Russia

**Keywords:** architectural C2H2 proteins, insulator, *gypsy*, Su(Hw), housekeeping genes, MADF-BESS transcriptional factors

## Abstract

Drosophila CP190 and CP60 are transcription factors that are associated with centrosomes during mitosis. CP190 is an essential transcription factor and preferentially binds to housekeeping gene promoters and insulators through interactions with architectural proteins, including Su(Hw) and dCTCF. CP60 belongs to a family of transcription factors that contain the N-terminal MADF domain and the C-terminal BESS domain, which is characterized by the ability to homodimerize. In this study, we show that the conserved CP60 region adjacent to MADF is responsible for interacting with CP190. In contrast to the well-characterized MADF-BESS transcriptional activator Adf-1, CP60 is recruited to most chromatin sites through its interaction with CP190, and the MADF domain is likely involved in protein–protein interactions but not in DNA binding. The deletion of the *Map60* gene showed that CP60 is not an essential protein, despite the strong and ubiquitous expression of CP60 at all stages of *Drosophila* development. Although CP60 is a stable component of the Su(Hw) insulator complex, the inactivation of CP60 does not affect the enhancer-blocking activity of the Su(Hw)-dependent *gypsy* insulator. Overall, our results indicate that CP60 has an important but redundant function in transcriptional regulation as a partner of the CP190 protein.

## 1. Introduction

In higher eukaryotes, two main variants of gene expression regulation can be distinguished [1,2,3,4]. In housekeeping genes, regulatory elements (enhancers) are located in close proximity to the promoter they control. Genes encoding regulators of development and cell specialization usually have complex regulatory regions that can contain up to several dozen specific enhancers, each of which regulates the expression of the target gene only in a certain group of cells for a precisely determined time. At present, the question of how interactions between enhancers and promoters located at distances sometimes reaching hundreds of kilobases are formed and regulated remains open [5,6,7,8]. It has recently been found that interactions between specialized regulatory elements, named tethering elements, can support remote interactions between enhancers and promoters [9,10,11,12]. Another class of regulatory elements, called insulators, is thought to be responsible for restricting the interaction between enhancers and promoters [13,14,15]. Insulators can function through direct interaction with enhancers and promoters or by forming chromatin loops that can block enhancer–promoter communication [13]. Insulators and arrays of housekeeping promoters also form a large part of the boundaries of topologically associated domains (TADs) in *Drosophila* [16,17]. According to current models, enhancer–promoter interactions within TADs are stimulated and, at the same time, suppressed between TADs [15]. Known *Drosophila* insulators are organized by architectural proteins, the characteristic feature of which are clusters consisting of five or more C2H2-type zinc-finger domains. Usually, 4–5 C2H2 domains separated by five amino acid (aa) linkers specifically bind to 12–15 bp DNA motifs [18].

The best-characterized Drosophila architectural proteins are Su(Hw), Pita, and dCTCF, which predominantly bind to insulators and gene promoters [19,20,21,22]. These architectural proteins interact with CP190, the 190 kDa (1096 aa) centrosome-associated protein, which binds predominantly to the promoters of housekeeping genes and insulator elements [23,24,25]. CP190 contains an N-terminal Broad-complex, Tramtrack, and Bric-à-brac (BTB) domain and a cluster of four C2H2-type zinc-finger domains in the central part of the protein [24,25]. CP190 is recruited to regulatory elements via interactions with the DNA-binding transcription factors [26,27,28,29,30].

The first identified partner of CP190 was CP60/Map60 (microtubule-associated protein 60), which contains a MADF (Myb/SANT-like domain in Adf-1) domain [31]. CP190 and CP60 are localized to the centrosome during mitosis [32,33] and to chromosomes during interphase [34,35]. CP190 begins to accumulate at the centrosome as soon as nuclear envelope breakdown occurs, whereas CP60 accumulates later and reaches maximal levels only in anaphase/telophase [32]. Despite the localization of CP190 and CP60 proteins on centrosomes during mitoses, their inactivation does not significantly affect the organization of microtubules or cell division [25,35]. However, CP190 inactivation leads to lethality at the larval pupa stages, and this protein is necessary for the activity of a large group of housekeeping promoters [27,34,35,36]. Several studies suggest that CP190 is involved in recruiting the EcR/Usp, NURF, DREAM, Nonstop identity, and SAGA complexes to the chromatin [37,38,39,40]. In the Bithorax complex, the CP190 protein binds to the boundaries that ensure independent activity of transcriptional domains [22,34,36,41].

CP60 also binds to chromatin [35], but its functional role in transcriptional regulation is unknown. By structure, CP60 belongs to a large family of transcription factors that contain a MADF domain. The MADF domain was first characterized in the transcriptional activator Adf-1 and is responsible for specific binding to the DNA motif and the protein–protein interactions necessary for the activation of transcription [42]. In some proteins, the MADF domains have lost the ability to bind to DNA and are involved only in protein–protein interactions [43]. The *Drosophila melanogaster* genome contains 55 genes encoding proteins with MADF domains [44]. The MADF family of transcription factors is hypothesized to be the result of gene duplications and divergence of functions over 40 million years ago [44], which led to redundancy and subfunctionalization of the activity of some MADF proteins. Some MADF proteins are involved in transcriptional activation, including Adf-1 [42,45,46] and Dorsal interacting protein 3 (Dip3) [47,48], and others in transcriptional repression, including Corepressor of Pangolin (Coop) [49] and Stonewall (stwl) [50,51]. At least 16 proteins possess a C-terminal BESS domain along with a MADF N-terminal domain [44]. The BESS domain is about 40 aa residues long and directs a variety of protein–protein interactions, including homodimerization, and is predicted to be composed of two alpha helices [52].

Here, we examine the functional role of CP60 as a new component of the Su(Hw) (suppressor of Hairy wing) insulator complex. The Su(Hw)-dependent insulators were among the first insulators found in higher eukaryotes [53,54,55,56,57] and are currently the best studied in *Drosophila* [13]. Su(Hw) forms a stable complex on chromatin with CP190, Mod(mdg4)-67.2 (modifier of *mdg4* 67.2) isoform, and HIPP1 (HP1 and insulator partner protein-1) [58,59,60,61,62]. The Su(Hw) complexes also interact with ENY2, Shep, and Rump, which are involved in recruiting transcriptional complexes and the regulation of insulator activity [13,63,64]. The Su(Hw) insulators can support distant interactions, form boundaries between active and repressed chromatin [13,56], repress promoters [65,66], and block enhancer–promoter interactions [53,67].

Here, we show that CP60 belongs to the MADF-BESS transcription factors and is a stable partner of CP190, but its functions are redundant. CP60 binds to all tested Su(Hw) sites but is not required for the enhancer-blocking activity of the *gypsy* insulator. Our results support the model that CP60 and unknown transcription factor(s) fulfill redundant functions in gene regulation.

## 2. Results

### 2.1. CP60 Is a Stable Component of the Su(Hw) Insulator Complex

**Figure 1:**
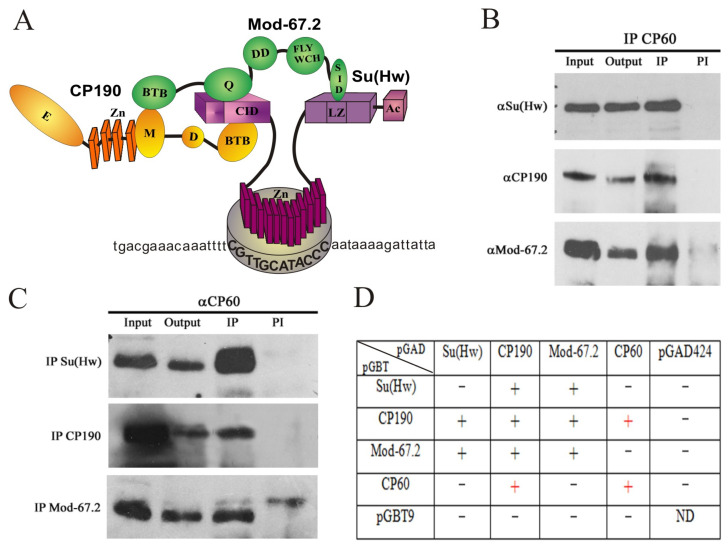
CP60 is a component of the Su(Hw) insulator complex: (**A**) Schematic presentation of the Su(Hw) insulator complex. The previously described interactions [58] between the components of the complex are shown. The CP190 domains are shown as yellow ovals, with four zinc fingers shown as yellow boxes; the Mod(mdg4)-67.2 domains are shown as green ovals; Su(Hw) domains are shown as lilac boxes. Domain abbreviations: CID—CP190 interacting domain; Ac—C-terminal acidic domain; Zn—zinc-finger domain; LZ—leucine zipper; BTB—BTB/POZ domain; Q—glutamine-rich region; DD—dimerization domain; FLYWCH—FLYWCH-type zinc finger; SID—Su(Hw) interacting domain; D—asparagine-rich domain; M—the microtubule- and centrosome-associated domain; E—glutamine-rich C-terminal domain. Bold capital letters indicate the Su(Hw) binding site. (**B**) Nuclear extract from *Drosophila* S2 cells was immunoprecipitated with antibodies against CP60, and the immunoprecipitates (IP) were analyzed via Western blotting for the presence of Su(Hw), Mod(mdg4)-67.2, and CP190 proteins. Input is the input fraction (1% of the lysate used for immunoprecipitation); output is the supernatant after immunoprecipitation; IP is the immunoprecipitate; PI is immunoprecipitation with nonspecific IgG. (**C**) Nuclear extract from *Drosophila* S2 cells was immunoprecipitated with antibodies against Su(Hw), Mod(mdg4)-67.2, or CP190, and the immunoprecipitates (IP) were analyzed via Western blotting for the presence of CP60 protein. The uncropped images for (**B**,**C**) are shown in Supplementary Materials Figure S2. (**D**) Identification of direct interactions between CP60 and other components of the Su(Hw) insulator complex using the yeast two-hybrid assay. The results are summarized in the table with + and—signs referring to a strong interaction or no interaction, respectively. Interactions with pGBT9 or pGAD424 vectors were used as the negative control. Interactions between CP190 and Mod(mdg4)-67.2 with Su(Hw) were used as a positive control.

The CP190 protein binds to many C2H2 architectural proteins, including Su(Hw) [27,30,59]. Since previous studies have shown an interaction between CP190 and CP60 [31], we hypothesized that CP60 might be included in the Su(Hw) complex, which consists of three main components, i.e., Su(Hw), CP190, and Mod(mdg4)-67.2 proteins (Figure 1A). We generated polyclonal rat antibodies against the 95–441 aa region of the CP60 protein and tested their specificity in S2 cells (Schneider 2 cells) (Supplementary Figure S1).

The interactions of CP60 with the components of the Su(Hw) complex were studied via coimmunoprecipitation in the nuclear extract of S2 cells. The CP60 antibodies precipitated Su(Hw), CP190, and Mod(mdg4)-67.2 proteins (Figure 1B). A reciprocal experiment testing immunoprecipitation of CP60 with antibodies against Su(Hw), CP190, or Mod(mdg4)-67.2 confirmed that CP60 is included in the Su(Hw) complex (Figure 1C).

To test the direct interactions of CP60 protein with components of the Su(Hw) complex, we used the yeast two-hybrid assay (Figure 1D). The cDNAs encoding full-length Su(Hw), Mod(mdg4)-67.2, CP190, and CP60 were fused with the GAL4-activating or DNA-binding domain. CP60 interacted in both experiments with only CP190 but not with Su(Hw) or Mod(mdg4)-67.2. In addition, the CP60 protein interacted with itself, which indicates the presence of a homodimerization domain in CP60, as in the MADF-BESS protein group in which the BESS domain is responsible for homodimerization [44,52].

### 2.2. CP60 Binds to the Su(Hw) Insulator Sites via Interaction with CP190

**Figure 2:**
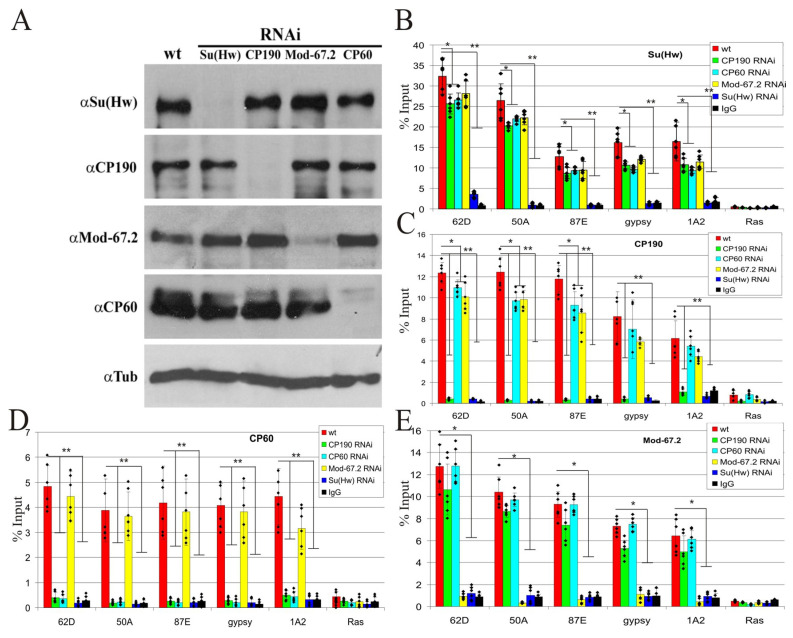
CP190 is responsible for CP60 recruitment to the Su(Hw) chromatin sites: (**A**) Western blot analysis of RNAi efficiency; wt—S2 cells without treatment; proteins indicated below the line (RNAi) were knocked out by RNAi; antibodies for staining are listed on the left of the panel. Anti-tubulin antibodies (αTub) were used as a loading control. The uncropped images are shown in Supplementary Materials Figure S3. (**B**–**E**) ChIP-qPCR analysis of binding (**B**) Su(Hw), (**C**) CP190, (**D**) CP60, and (**E**) Mod(mdg4)-67.2 proteins to the selected Su(Hw) sites in wild-type (wt) S2 cells and after RNAi inactivation of each protein. The *ras64B* coding region (Ras) was used as a control that does not contain Su(Hw) binding sites. IgG—immunoprecipitation with nonspecific IgG. The percentage recovery of immunoprecipitated DNA (Y axis) was calculated relative to the amount of input DNA. Error bars indicate SDs of quadruplicate PCR measurements from two independent biological samples of chromatin. Asterisks indicate significance levels: * *p* < 0.05 and ** *p* < 0.01 (Student’s *t*-test). Dots on the bar plots indicate the values of individual experiments.

Our results showed that CP60 interacts only with the CP190 component of the Su(Hw) complex in vitro. To test the role of the interaction between CP60 and CP190 in recruitment to the Su(Hw) sites, we knocked down the Su(Hw), CP190, CP60, and Mod(mdg4) proteins by RNAi in S2 cells and examined the proteins’ binding ability by ChIP-qPCR with best-characterized intergenic Su(Hw) binding regions: 62D (sites); 50A (two sites); 87E (two sites); 1A2 region (two sites) located in the 3′ region of the *yellow* gene; and *gypsy* (twelve sites)—the insulator region from the *gypsy* retrotransposon (Figure 2). These regions were characterized in several previous studies as strong Su(Hw) binding sites [60,68]. The efficiency of RNAi treatment was confirmed via Western blotting (Figure 2A). The knock-down of either CP60, CP190, or Mod(mdg4)-67.2 decreased the binding of Su(Hw) (Figure 2B). This suggests a possible role for these proteins in stabilizing Su(Hw) binding to its target sites. The binding of CP190 was dependent on Su(Hw) but was not affected by CP60 or Mod(mdg4)-67,2 knock-down (Figure 2C). CP60 did not bind to the tested sites when either Su(Hw) or CP190 was knocked down (Figure 2D). Finally, Mod(mdg4)-67.2 binding depends only on Su(Hw) (Figure 2E). These results indicate that CP60 binds to CP190 and is recruited to the Su(Hw) sites through the Su(Hw)–CP190 interaction.

### 2.3. The MADF Domain of CP60 Does Not Interact with the Su(Hw) Binding Regions

**Figure 3:**
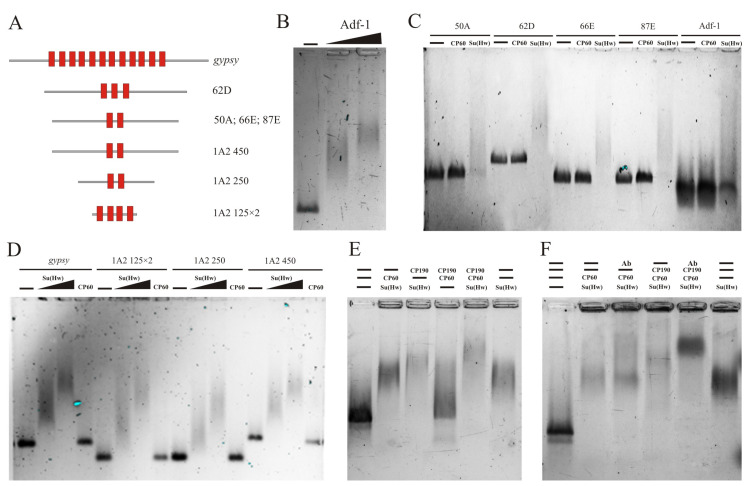
Analysis of the in vitro association between Su(Hw) or CP60 and Su(Hw) motifs: (**A**) Schematic representation of the tested Su(Hw) binding sites. The red squares show the localization of the Su(Hw) motifs. (**B**) The Adf-1 binding site from *bxd* PRE and purified Adf-1 protein were used in EMSA as the positive control. DNA fragments without protein are marked with a minus (−) sign. The triangle represents a threefold increase in Adf-1 concentration from 0.05 µg to 0.15 µg. (**C**) EMSA of the binding of Su(Hw) (0.05 µg) and CP60 (0.05 µg) proteins to DNA fragments 50 A, 62 D, 66 E, and 87 E. The Adf-1 binding site was used as the negative control for the Su(Hw) or CP60 proteins. (**D**) EMSA of the binding of CP60 and Su(Hw) proteins to the *gypsy* insulator (*gypsy*) and two copies of the minimal 125 bp 1A2 region (1A2 125 × 2), and 250 bp (1A2 250) and 450 bp (1A2 450) regions of 1A2 insulator site. The amount of Su(Hw) protein was increased threefold from 0.05 µg to 0.15 µg. Other designations are as in (**B**). (**E**) Cooperation of Su(Hw), CP190, and CP60 in the binding to the *gypsy* insulator. The combination of proteins (0.05 µg) is indicated above in the panel. The absence of the corresponding protein in the EMSA probe is marked as a minus (−) sign. (**F**) EMSA supershift by CP60 antibodies and different combinations of Su(Hw) insulator proteins (0.05 µg). Ab—CP60 antibodies were added to the probe. Other designations as in (**E**).

Figure 2B shows that the reduction expression of CP60 affects the binding of Su(Hw) to the tested sites. Since MADF is assumed to be a DNA-binding domain [47,69], it is possible that CP60 stabilizes the Su(Hw) complex on chromatin by binding the MADF domain to sequences located near the Su(Hw) motifs. To test this, we examined the binding of in vitro-prepared CP60 protein in electrophoretic mobility shift assay (EMSA) (Figure 3). We used Su(Hw) binding sites (Figure 3A), including Su(Hw) motifs, located in the center of 250–300 bp DNA fragments. As a positive control, we used a DNA fragment from *bxd* PRE (polycomb response element) containing motifs to which the MADF domain of the Adf-1 protein specifically binds [70]. In the control experiment, the Adf-1 protein was successfully bound to the consensus sequence (Figure 3B). The CP60 protein was not able to bind to any of the tested sequences, while Su(Hw) bound efficiently, with a shift that increased with higher Su(Hw) concentrations (Figure 3C,D).

To further investigate the interdependence of the binding of the studied proteins, we used the EMSA supershift method. We analyzed the change in the mobility of the *gypsy* insulator fragment, consisting of 12 Su(Hw) binding sites, in combination with various insulator proteins (Figure 3E). No shift was observed if only CP60 and CP190 were added without Su(Hw), which confirms that CP60 and CP190 are not able to bind to the insulator sequence. The combined addition of Su(Hw) and CP60 proteins resulted in a mobility shift but only to the same extent as the Su(Hw) protein alone. The addition of Su(Hw) and CP190 resulted in a more intense mobility shift than the Su(Hw) protein alone. Thus, only CP190, and not CP60, is able to interact with Su(Hw) bound to the *gypsy* insulator. The addition of all three proteins resulted in a more effective shift than in the case of Su(Hw) and CP190 (Figure 3E).

The results were confirmed using a supershift experiment with antibodies to the CP60 protein (Figure 3F). When adding CP60 antibodies to the Su(Hw) and CP60 proteins, a shift was observed at the same level as that with the Su(Hw) protein alone, while the addition of antibodies to the three proteins caused a significant supershift compared with the control. These experiments indicate that the CP60 protein is recruited into the insulator complex only through interaction with the CP190 protein.

### 2.4. Mapping CP60 Domains Involved in the Interaction with CP190 and Homodimerization

**Figure 4:**
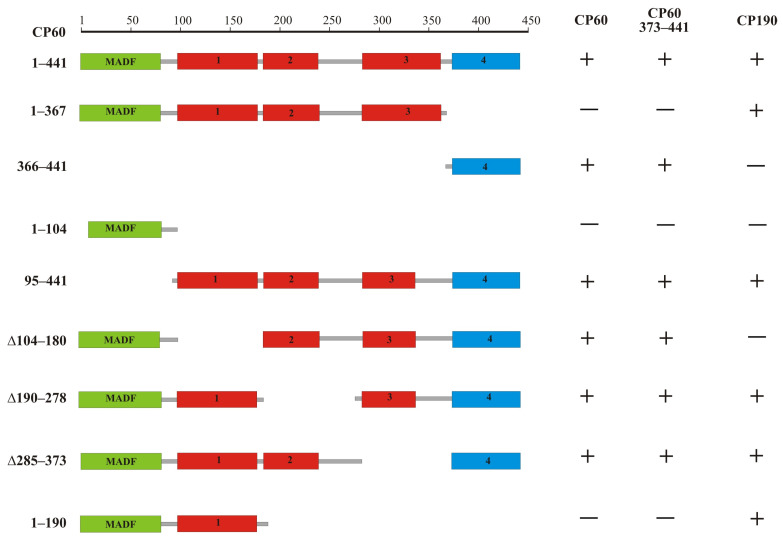
Mapping domains of CP60 that are responsible for homodimerization and interaction with CP190 in the yeast two-hybrid assay. The scale at the top of the figure is in amino acids. A schematic representation of the CP60 protein is shown with deletion margins on the left. The MADF domain is shown as a green rectangle. Conserved regions 1–3 are represented by red rectangles. The fourth C-terminal conserved region is shown as a blue rectangle. The interactions are summarized in columns on the right-hand side with + and − referring to the presence and absence of interactions, respectively.

The CP60 protein is highly conserved not only in *Drosophilidae* (Supplementary Figure S4) but also in many species of *Diptera* (Supplementary Figure S5). Along with the predicted MADF domain (1–84 aa), there are four conserved regions in the CP60 protein. To map regions in CP60 that are required for homodimerization and the interaction with CP190, we used the yeast two-hybrid assay (Figure 4). cDNAs encoding truncated variants of CP60 were fused with the GAL4 DNA-binding domain. The complete cDNAs of CP190 and CP60 were fused with the GAL4 activation domain. CP190 interacted with the first conserved region (104–189 aa), which is located adjacent to MADF, and CP60 interacted with the conserved C-terminal region (337–441 aa) (Figure 4). To test whether only the C-terminal domain is responsible for homodimerization, the GAL4 activation domain was fused with the C-terminal 373–441 aa. The 337–441 aa region was sufficient for homodimerization in the yeast two-hybrid assay.

**Figure 5:**
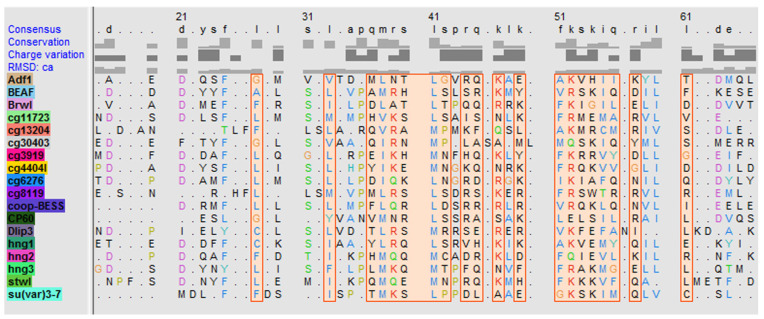
Multiple alignment analysis of the CP60 C-terminal conserved region (440–441 aa) sequence with different known BESS domains. Alignment regions are shown as colored boxes or outlines that enclose one or more residue symbols. The residue coloring corresponds to the Clustal X color code (Supplementary Table S1).

Like other transcription factors of the MADF-BESS family [44], CP60 contains an N-terminal MADF domain and a homodimerizing C-terminal region. We supposed that CP60 belongs to this group of transcription factors and, to test this, we performed multiple alignment analyses of the C-terminal domain of CP60 (373–441 aa) with known BESS domains and obtained a potential domain structure using the alphafold program. The 440–441 aa region of CP60 displays a similar structural organization as the BESS domain (Supplementary Figure S6A) and this region is predicted to form dimers with high probability (>70%; Supplementary Figure S6B). Multiple alignment analysis revealed significant sequence similarity or identity for the BESS domain, indicating that the proteins have a common evolutionary origin (Figure 5), and thus it is likely that CP60 belongs to the group of MADF-BESS transcription factors.

### 2.5. CP60 Is a Redundant Protein

**Figure 6:**
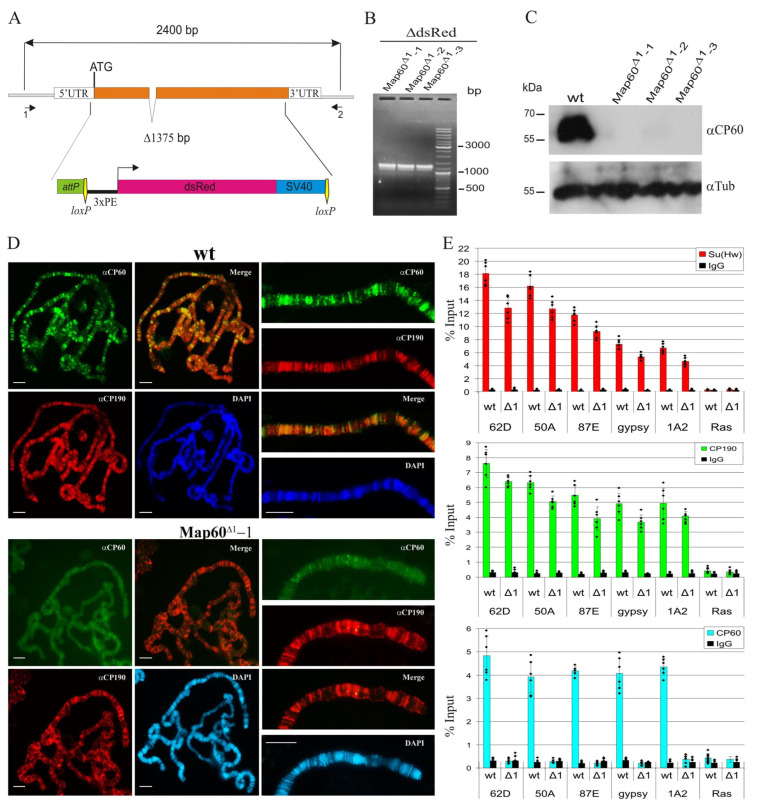
Functional analysis of the *Map60* gene: (**A**) CRISPR/Cas9 deletion of the *Map60* gene. The CP60 coding regions are shown as orange boxes. White rectangles represent the 5′ and 3′UTR. CRISPR targets are shown as vertical bars. Primer sequences used in the CRISPR/Cas9 genome editing are given in Supplementary Table S2. The *Red* reporter (magenta box), controlled by the *3xP3* promoter (black line), was used for the selection of the *Map60* deletion. The *attP* and *lox* sites were used for genome manipulation and are shown as a green box and vertical yellow arrows, respectively. SV40 terminator is shown as a blue box. The two black arrows represent primers 1 and 2, which were used to test the obtained mutations using PCR. (**B**) PCR analysis of genomic DNA from *Map60^Δ1^* lines after *CRE/loxP* excision of marker *dsRed* cassette (*ΔdsRed*). The molecular weight in bp is shown on the right. (**C**) Western blot analysis (8% SDS PAGE) of protein extracts prepared as described previously [72] from adult three-day-old males of the wild-type (wt) line and three lines homozygous for *Map60^Δ1^*. The membrane was sequentially stained with tested polyclonal rat antibodies against CP60 (αCP60) and antibodies against tubulin (αTub) as loading control. The molecular weight in kDa is shown on the left. The uncropped images are shown in Supplementary Materials Figure S7. (**D**) Polytene chromosomes from the salivary glands of third-instar *y^2^w^1118^ct^6^* (wt) and *y^2^w^1118^ct^6^*; *Map60^Δ1^-1/Map60^Δ1^*-1 (Map60^Δ1^-1) larvae costained with rat anti-CP60 antibodies (green), rabbit anti-CP190 antibodies (red), and DAPI (blue) Scale bars, 10 μm. (**E**) ChIP-qPCR analysis of Su(Hw), CP190, and CP60 binding to Su(Hw) sites (description in Figure 2) in wt and Map60^Δ1^-1 (Δ1) lines. The *ras64B* coding region (Ras) that did not contain Su(Hw) binding sites was used as a control. IgG—immunoprecipitation with nonspecific IgG. The percentage recovery of immunoprecipitated DNA (Y axis) was calculated relative to the amount of input DNA. Error bars indicate SDs of quadruplicate PCR measurements from two independent biological samples of chromatin. Significance levels: *p* < 0.05 (Student’s *t*-test). Dots on the bar plots indicate the values of individual experiments.

According to FlyBase, the *Map60* gene is strongly expressed during all stages of *Drosophila* development and in all tissues suggesting that CP60 has an important function in transcriptional regulation. To test the functional role of CP60 in vivo, we generated null *Map60* alleles using CRISPR/Cas9-based genome editing [71]. We used a two-step genome engineering platform that combines CRISPR and the *φC31/attP* recombination system [71]. A 1375 bp region of the *Map60* gene from the 5′UTR to the end of the second exon (2R:9591007…9592382, FlyBase, r6.52) was substituted with the *attP* site next to a *3P3:Red* reporter flanked by *lox* sites (Figure 6A). We obtained three independent lines with the null *Map60^Δ1^* mutation, which were confirmed by PCR (Figure 6B). *Map60^Δ1^* flies have normal viability and fertility and do not have a mutant phenotype. Western blot analysis with the control *y^2^w^1118^ct^6^* (wt) line and three lines carrying the *Map60^Δ1^* allele showed that antibodies specifically recognize a 60 kDa band only in the wild-type line (Figure 6C). We also tested the CP60 antibodies in vivo by immunostaining the polytene chromosomes from three instar larvae of the wild-type and *Map60^Δ1^* lines. We observed more than 200 bands on the wild-type polytene chromosomes, suggesting that CP60 binds to chromosomes, while we did not see bands on the *Map60^Δ1^* polytene chromosomes, thus confirming the specificity of the CP60 antibodies (Figure 6D). Finally, we compared the enrichment of Su(Hw) and CP190 proteins in the wild-type and *Map60^Δ1^* pupae. As expected, in the *Map60^Δ1^* background the CP60 protein was absent at the Su(Hw) binding sites, and enrichment for the Su(Hw) protein and, accordingly, for the CP190 protein was slightly reduced compared with the wild type (Figure 6E).

To directly test the role of CP60 in the insulator function, we used the previously characterized *y^2^* and *ct^6^* mutations, which are induced by inserting the *gypsy* element between enhancers of the *yellow* and *cut* genes, respectively [59]. The *gypsy* insulator blocks the enhancers and results in mutant phenotypes. The *Map60^Δ1^* mutation did not affect the phenotypes of the *y^2^* and *ct^6^* alleles. Next, we used a system based on the inactivation of Mod(mdg4)-67.2 by a *mod(mdg4)^u1^* mutation. In the absence of Mod(mdg4)-67.2, the *gypsy* insulator only weakly blocks the *cut* enhancer and acts as a silencer that represses *yellow* expression [60]. We combined *mod(mdg4)^u1^* with *Map60^Δ1^* and *y^2^* or *ct^6^* mutations. No phenotypic changes were observed, indicating that CP60 does not affect the activity of the *gypsy* insulator even in the absence of the Mod(mdg4)-67.2 protein. Together, our results suggest that CP60 functions are redundant, and its inactivation can be potentially substituted by other members of the MADF-BESS transcription factor family.

### 2.6. CP190 Is the Main Partner of CP60 at Chromatin Sites

**Figure 7:**
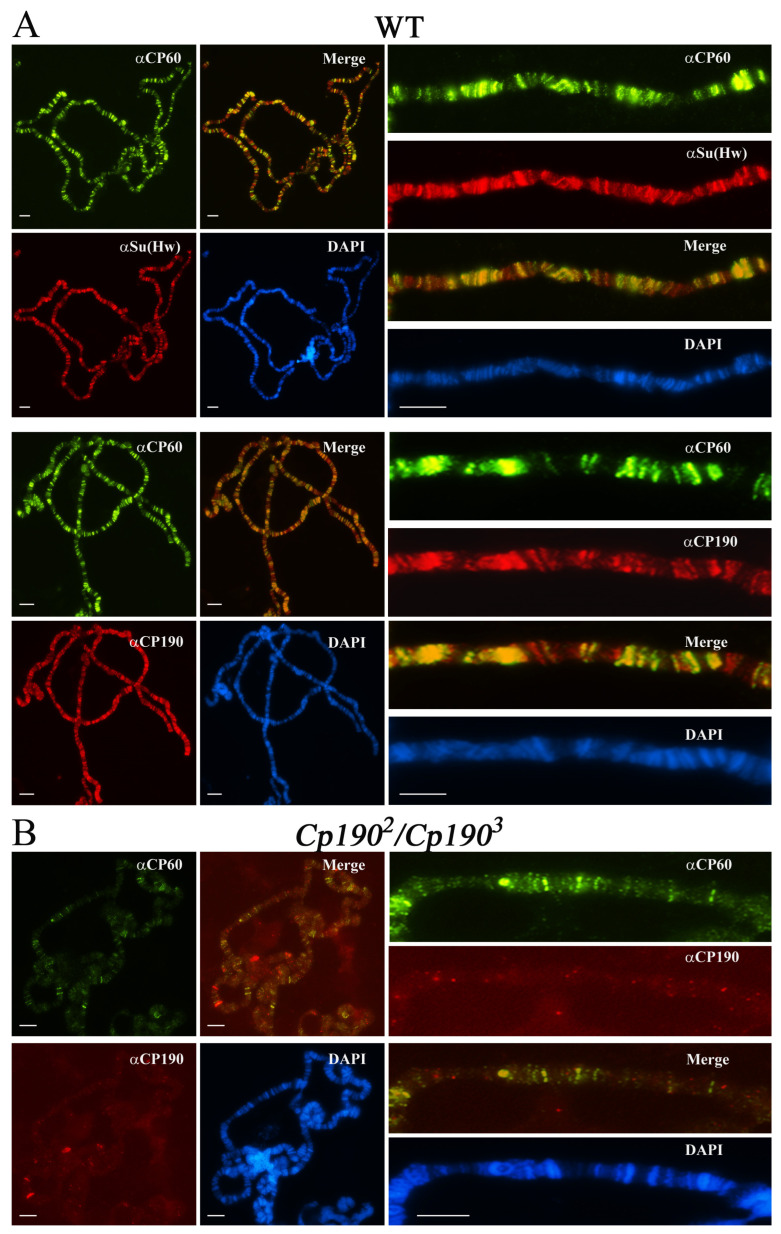
Testing the genome-wide distribution of CP60 and its colocalization with the components of the Su(Hw) complex. Polytene chromosomes from the salivary glands of third-instar (**A**) *y^2^w^1118^ct^6^* (wt) and (**B**) *y^2^w^1118^ct^6^*; *Cp190^2^/Cp190^3^* (*Cp190^2^/Cp190^3^*) larvae costained with rat anti-CP60 antibodies (green), rabbit anti-CP190 or anti-Su(Hw) antibodies (red), and DAPI (blue). Scale bars, 10 μm.

To confirm the genome-wide colocalization of CP60 and CP190 in vivo, we stained polytene chromosomes with antibodies to CP60, CP190, and Su(Hw) proteins. CP60 colocalized with Su(Hw) and CP190 at most sites (Figure 7A), confirming that CP60 is a component of the Su(Hw) complexes. However, CP60 also colocalized with CP190 at non-Su(Hw) sites, suggesting that it participates in other CP190-dependent complexes. These results are consistent with the model that CP190 is the main CP60 recruiter on chromatin. To further test this, we compared the binding of CP60 to polytene chromosomes of wild-type and *Cp190^2^/Cp190^3^* (CP190 null background) larvae (Figure 7B). No bands were observed on polytene chromosomes labeled with CP190. Accordingly, the number of bands marked by CP60 also sharply decreased. In contrast, on polytene chromosomes *Cp190^2^/Cp190^3^*, there was only a slight decrease in Su(Hw) binding, which is consistent with the observed role of CP190 in the recruitment of Su(Hw) to chromatin. Together, these results confirm the key role of CP190 in the recruitment of CP60 to the Su(Hw) sites and other CP190 binding regions but do not exclude the association of CP60 with other proteins or binding to DNA through the MADF domain at a small number of sites.

**Figure 8:**
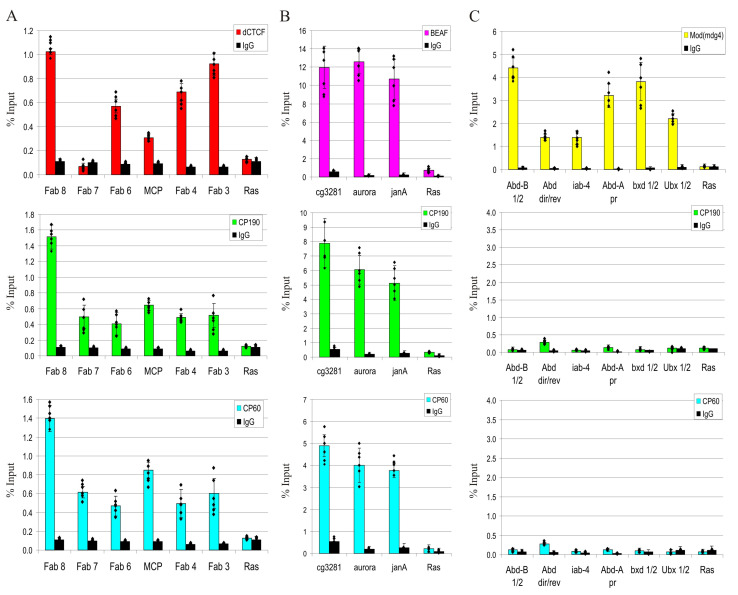
CP190 is responsible for CP60 recruitment to dCTCF/CP190 and BEAF/CP190 sites. ChIP-qPCR analysis of binding of dCTCF, CP190, and CP60 to the (**A**) dCTCF (Fab3, Fab4, Fab8, and MCP)- and Pita (Fab7)-dependent sites of the Bitorax complex. (**B**) BEAF-dependent sites from promoter regions of the *aurora*, *cg3281*, and *janA* genes. (**C**) Promoter and regulatory regions from the Bitorax complex are not bound by CP190 but are enriched in isoforms of the Mod(mdg4) protein. The *ras64B* coding region (Ras) was used as a control devoid of dCTCF/CP190- and BEAF/CP190 binding sites. IgG—immunoprecipitation with nonspecific IgG. The percentage recovery of immunoprecipitated DNA (Y axis) was calculated relative to the amount of input DNA. Error bars indicate SDs of quadruplicate PCR measurements from two independent biological samples of chromatin. Significance levels: *p* < 0.01 (Student’s *t*-test). Dots on the bar plots indicate the values of individual experiments.

To confirm that CP60 is a general partner of CP190 at most chromatin sites, we tested the binding of CP60 to several dCTCF/CP190 and CP190/BEAF sites in S2 cells and observed that the CP60 protein binds to all tested sites (Figure 8A,B). The presence of CP60 at the sites depends on the presence of the CP190 protein, as no enrichment of the CP60 protein was observed on sites enriched in Mod(mdg4) proteins but not containing the CP190 protein (Figure 8C). Together, our results indicate that CP190 is the main recruiter of CP60 to chromatin.

## 3. Discussion

Here, we show that the conserved CP60 protein belongs to the insect-specific family of MADF-BESS transcription factors [44]. The best-characterized member of this group is Adf-1, a transcription activator involved in the stabilization of the TFIID complex on promoters. Adf-1 was first identified as a promoter-binding factor that activated the alcohol dehydrogenase (Adh) gene [69] and later was reported to bind to promoters genome-wide [70]. The Adf-1 protein participates in recruiting the Mediator complex to chromatin and the Adf-1 interactome includes Mod(mdg4) and BEAF architectural proteins [73].

The MADF domain of Adf-1 has two functions: It binds to a specific DNA motif in promoter regions and is required for the recruitment of transcription complexes [74]. We did not find any DNA-binding activity of CP60. It is possible that the MADF domain of CP60 has evolved toward protein–protein interactions and is specialized toward the recruitment of transcriptional complexes.

It was previously shown that CP60 protein colocalizes with CP190 on the centrosome during mitosis [32,33]. However, these proteins bind to the centrosome independently and CP190 and CP60 accumulate at the centrosome at the early and late mitosis stages, respectively [34,35]. In contrast, CP190 and CP60 tightly interact in the interphase nucleus. The CP60 conserved region (104–189 aa) adjacent to the MADF domain is responsible for the interaction with CP190. CP190 is critical for the activity of most promoters that determine the expression of housekeeping genes and is involved in the functional activity of insulators. CP190 functions in cooperation with Z4/Putzig and Chromator, which are essential proteins [34]. It was suggested that CP190/Z4/Chromator are required for recruiting of transcriptional complexes like NURF and DREAM [37,38], and are the main transcriptional factors to promoters [39]. CP190 is recruited to regulatory elements via interactions with a large group of architectural proteins, including the Su(Hw) protein. The Su(Hw) protein is responsible for the activity of the strongest *Drosophila* insulator located in the *gypsy* retrotransposon, but the CP60 protein is not essential for the insulator activity of the Su(Hw) complex. Other architectural proteins preferentially recruit CP190 to gene promoters independently of the Su(Hw) protein [34,75,76].

CP60 is highly expressed and recruited to chromatin through interactions with CP190 at most of its genomic sites. All these observations indicate that CP60 plays an important role in the activation of CP190-dependent transcription at promoters as has been shown for Adf1 [69,70,73]. However, the deletion of the *Map60* gene shows that CP60 is not an essential protein. It seems likely that other transcription factors of the MADF-BESS family functionally substitute for the absence of the CP60 protein. Such redundancy was previously described for MADF-BESS hinge1, hinge2, and hinge3 proteins involved in the Wg signaling pathway [44] and for Stonewall (Stwl) and Brickwall (Brwl) proteins, which are required for maintaining the female germline [77]. Also, Stwl colocalizes with binding sites for BEAF-32, ZIPIC, Pita, and Su(Hw) proteins, which recruit CP190 to chromatin in S2 cells [78] and with CP190 on larvae polytene chromosomes [79]. Further studies are needed to understand which transcription complexes are recruited by CP60 and to identify MADF-BESS proteins that have similar functions to CP60. This will allow for a better understanding of the mechanisms of the formation of active promoters as a result of the recruitment of CP190 and its partners.

## 4. Materials and Methods

### 4.1. Plasmids and Cloning

To generate pSK-CP60 plasmid, CP60 cDNA was amplified with primers 5′-ataGAATTCatggcaatccaactggac-3′ (upstream, containing EcoRI site) and 5′-ataGGATCCctgctccagttcgtcg-3′ (downstream, containing BamHI site) from cDNA library. The PCR product was cleaved with BamHI and EcoRI, and the 1330 bp BamHI–EcoRI fragment was cloned into pBluescript II SK + vector cleaved with the same enzymes.

Plasmids for the yeast two-hybrid assay were prepared using the full-sized and truncated versions of CP60 protein, CP190, Mod(mdg4)-67.2, and Su(Hw) fused with pGAD424 and pGBT9 vectors (Clontech, Mountain View, CA, USA). Details of the cloning procedures are described in the Supplementary Materials.

Plasmid for expression CP60 protein in *E. coli* was generated by cloning the BamHI (filled in with Klenow fragment)–EcoRI fragment from pSK-CP60 into pET32a cleaved with NotI (filled in with Klenow fragment) and EcoRI. Expression constructs for Su(Hw) and CP190 proteins were described previously [58,59,60,66]. Plasmid for expression Adf1 protein was kindly provided by Dr. Maxim Erokhin [80].

### 4.2. RNA Interference in Drosophila S2 Cells

Individual DNA fragments of approximately 700 bp, containing coding sequences for the proteins to be “knocked out”, were amplified via PCR using the primers contained a T7 RNA polymerase binding site (taatacgactcactatag) at the 5′ end (Supplementary Table S3). PCR products were purified using a gel extraction kit (Zymo Research, Irvine, CA, USA) as recommended by the manufacturer. The purified PCR products were used to produce double-stranded RNA (dsRNA) using a Megascript T7 transcription kit (Ambion, Austin, TX, USA). The RNA was purified according to the manufacturer’s protocol, heated at 65 °C for 30 min, and left to cool at room temperature. Six micrograms of dsRNA were analyzed using 1% agarose gel electrophoresis to ensure that the majority of the dsRNA existed as a single band of approximately 700 bp. The S2 cells were cultured in an SFX medium (HyClone) in 30 mm Petri dishes at 25 °C. RNAi experiments were performed when the culture reached a density of ~1 × 10^6^ cells/mL. dsRNA was directly added to the media to a final concentration of 37 nM. For dsRNAs of approximately 700 bp, this corresponds to 15 μg of dsRNA. On day 3 after the first dsRNA treatment, the cells were used for subsequent experiments.

### 4.3. Immunoprecipitation Assay

Drosophila S2 cells were grown in SFX medium (HyClone, Logan, UT, USA) at 25 °C. The S2 cells were cultured as described [81]. Cell lysate preparation and immunoprecipitation are described in the Supplementary Materials. Rabbit antibodies against CP190 (1:500), Su(Hw) (1:1000), Mod(mdg4)-67.2 (1:200), and rat antibodies against CP60 (1:300) were used for immunoprecipitations. The results were analyzed via Western blotting. Proteins were detected using the ECL Plus Western Blotting substrate (Thermo Scientific, Rockford, IL, USA) with anti-CP190 (1:5000), anti-Su(Hw) (1:3000), anti-Mod(mdg4)-67.2 (1:2000), and anti-CP60 (1:3000) antibodies.

### 4.4. Yeast Two-Hybrid Assay

The yeast two-hybrid assay was performed with plasmids and protocols from Clontech (Palo Alto, CA, USA). For growth assays, plasmids were transformed into yeast strain pJ69-4A using the lithium acetate method, as described by the manufacturer, and plated on media without tryptophan and leucine. After 3 days of growth at 30 °C, the cells were plated on selective media without tryptophan, leucine, histidine, and adenine, and their growth was compared after 2–3 days. Each assay was repeated three times.

### 4.5. Chromatin Preparation and ChIP Analysis

Chromatin was prepared from the S2 cells and middle pupa stage, and the resulting chromatin preparation was used for ChIP experiments, as described previously [82]. Immunoprecipitated DNA was quantified using qPCR, with SYBR green (Bio-Rad Cat# 170-8882). Primers were positioned in the middle of the binding region, as identified in ModEncode by ChIP-seq. The primer sequences used in PCR for ChIP analyses are shown in Supplementary Table S4. Analyses were performed on three biological replicates. Rabbit antibodies against CP190 (1:500), Su(Hw) (1:1000), Mod(mdg4)-67.2 (1:200), mouse antibodies against common part of Mod(mdg4) protein (1:500), and rat antibodies against CP60 (1:300) were used for immunoprecipitations.

### 4.6. Protein Expression and Purification

CP60, Su(Hw), CP190, and Adf-1 proteins were expressed as fusions with 6xHis tag in the pET32a vector (Novagen). *Escherichia coli* BL21 cells were transformed with plasmids expressing these proteins and were grown in LB media to an *A*_600_ of 1.0 at 37 °C and then induced with 1 mM IPTG at 18 °C overnight. ZnCl_2_ was added to the final concentration of 0.2 mM before induction. The cells expressing recombinant proteins were pelleted at 5000 *g* for 15 min, resuspended in 1–2 mL of buffer A (50 mM NaH_2_PO4 pH = 8.0, 300 mM NaCl, 10 mM imidazole, 1 mM PMSF) and disrupted via sonication. The lysate was cleared via centrifugation at 15,000 *g* for 15 min, and the supernatant was incubated with Ni Sepharose (GE Healthcare, Uppsala, Sweden) in the same buffer. After washing three times with wash buffer (50 mM NaH_2_PO4 pH = 8.0, 300 mM NaCl, 20 mM imidazole), the bound proteins were eluted with elution buffer (50 mM NaH_2_PO4 pH = 8.0, 300 mM NaCl, 250 mM imidazole). After elution, the Roche Protease Inhibitor Cocktail 11836145001 was added.

### 4.7. Electrophoretic Mobility Shift Assay (EMSA)

DNA fragments were labeled using PCR with primers containing the Cy5 fluorophore (Evrogen, Moscow, Russia). The sequences of tested Su(Hw) binding sites generated via PCR are given in Supplementary Table S5. Samples (20 µL) containing 60–80 ng of Cy5-labeled DNA fragments were incubated with 0.05 or 1.2 μg of purified recombinant proteins for 30 min at room temperature with added 1 ng Poly(dI-dC) as the competitor. Incubation was performed in 1 × PBS (pH 8.0) containing 5 mM MgCl_2_, 0.1 mM ZnSO_4_, 1 mM DTT, 0.1% NP-40 and 10% glycerol. The mixtures were resolved in 1% agarose gel in 1 × TBE buffer at 5 V/cm. Gels were scanned on the ChemiDoc MP Imaging system (Bio-Rad, Hercules, CA, USA).

### 4.8. Immunostaining of Polytene Chromosomes

*Drosophila* 3rd instar larvae were cultured at 18 °C under standard conditions. Polytene chromosome staining was performed as described [83]. The following primary antibodies were used: anti-CP190 (rabbit) 1:300, anti-Su(Hw) (rabbit) 1:300, and anti-CP60 (rat) 1:200. The applied secondary antibodies were Cy3-AffiniPure Donkey Anti-Rabbit 1:200 and FITC-AffiniPure Donkey Anti-Rat 1:200 (Jackson Immuno Research, West Grove, PA, USA). The polytene chromosomes were costained with 4′,6-diamidino-2-phenylindole (DAPI) (AppliChem, Darmstadt, Germany).

Analysis was performed with a Zeiss fluorescence microscope (Axio Observer.Z1, Jena, Germany), which integrated an OptiGrid Structured Illumination Microscopy system (Qioptiq, Luxembourg). Fiji was used to process images.

### 4.9. Antibodies

Antibodies against CP60 (95 aa–441 aa) were raised in rats and purified from the sera via ammonium sulfate fractionation followed by affinity purification on CNBr-activated Sepharose (GE Healthcare, Uppsala, Sweden) according to standard protocols. Affinity-purified antibodies were tested with immunoblot analysis (Supplementary Figure S1). Antibodies to Su(Hw), CP190, Mod(mdg4)-67.2, and the common part of Mod(mdg4) were generated in our laboratory and described previously [84]. Antibody to BEAF was kindly provided by Dr. Maxim Eroknin (IGB RAS, Moscow, Russia).

### 4.10. Germ-Line Transformation, Genetic Crosses, and Phenotypic Analysis

All flies were maintained at 25 °C, on standard yeast medium. To obtain flies with deletion in the *Map60* gene, the DNA of reporter constructs was injected into preblastoderm embryos with the y^1^ M{w^+mC^ = nos−Cas9.P}ZH-2A w* genotype [71]. The resulting flies were crossed with *y^2^ sc^D1^w^1118^ ct^6^* flies, and the progeny carrying the *Map60^Δ1^* mutation was identified using *dsRed* expression.

The lines with excisions of *3xPE:dsRed* were obtained by crossing flies carrying the *Map60^Δ1^* mutation with the Cre recombinase-expressing line *y^1^w^1118^*; *CyO*, *P[w^+^,cre]/Sco*; *+.* All excisions were confirmed through PCR analysis.

To test the effects of CP60 protein on *yellow* gene expression, *y^2^ sc^D1^w^1118^ ct^6^*; *Map60^Δ1^-1/ Map60^Δ1^-1*; *+* line were crossed into the *mod(mdg4)^u1^/mod(mdg4)^u1^* mutant background, as described previously [60].

The effects of *Map60^Δ1^* mutation on insulator function were scored by two researchers independently. The level of expression of *yellow*, *scute*, and *cut* phenotypes was evaluated in 3–5-day-old males developing at 25 °C, using as reference the *y^2^sc^D1^w^1118^ct^6^* flies. At least 100 flies were scored.

## Data Availability

All data generated or analyzed during this study are included in this published article and its Supplementary Materials.

## Supplementary Materials

The following supporting information can be downloaded at: https://www.mdpi.com/article/10.3390/ijms241915029/s1.
