# Supplementary material for: The MADF-BESS Protein CP60 Is Recruited to Insulators via CP190 and Has Redundant Functions in Drosophila
Source: Int J Mol Sci. 2023 Oct 9;24(19):15029. doi: 10.3390/ijms241915029 (PMC10573801; doi:10.3390/ijms241915029)
Supplement: Supplementary file 1 [file ijms-24-15029-s001.zip › ijms-2602100-supplementary.pdf]

Supplementary information for

The MADF-BESS Protein CP60 Is Recruited to Insulators via CP190 and Has Redundant Functions in *Drosophila*

Larisa Melnikova[1], Varvara Molodina[1], Valentin Babosha[2,3], Margarita Kostyuchenko[1], Pavel Georgiev[2], Anton Golovnin[1*]

[1]Department of Drosophila Molecular Genetics, Institute of Gene Biology, Russian Academy of Sciences, 34/5 Vavilov St., 119334, Moscow, Russia; lsm73@mail.ru (L.M.); molodina_varvara@mail.ru (V.M); rita_k@mail.ru (M.K.); agolovnin@mail.ru (A.G)

[2]Department of the Control of Genetic Processes, Institute of Gene Biology, Russian Academy of Sciences, 34/5 Vavilov St., Moscow 119334, Russia; v.babosha@gmail.com (V.B.); georgiev_p@mail.ru (P.G.)

[3]Center for Precision Genome Editing and Genetic Technologies for Biomedicine, Institute of Gene Biology, Russian Academy of Sciences, 34/5 Vavilov St., Moscow 119334, Russia; v.babosha@gmail.com (V.B.)

*Corresponding author:

E-mail: agolovnin@mail.ru (AG)

Table S1. Clustal X Default Colouring

Clustal X Default Colouring			
**Category**	**Colour**	**Residue at position**	**{ Threshold, Residue group }**
Hydrophobic	BLUE	A,I,L,M,F,W,V	{>60%, WLVIMAFCHP}
		C	{>60%, WLVIMAFCHP}
Positive charge	RED	K,R	{>60%,KR},{>80%, K,R,Q}
Negative charge	MAGENTA	E	{>60%,KR},{>50%,QE},{>85%,E,Q,D}
		D	{>60%,KR}, {>85%, K,R,Q}, {>50%,ED}
Polar	GREEN	N	{>50%, N}, {>85%, N,Y}
		Q	{>60%,KR},{>50%,QE},{>85%,Q,E,K,R}
		S,T	{>60%, WLVIMAFCHP}, {>50%, TS}, {>85%,S,T}
Cysteines	PINK	C	{>85%, C}
Glycines	ORANGE	G	{>0%, G}
Prolines	YELLOW	P	{>0%, P}
Aromatic	CYAN	H,Y	{>60%, WLVIMAFCHP}, {>85%, W,Y,A,C,P,Q,F,H,I,L,M,V}
Unconserved	WHITE	any / gap	If none of the above criteria are met

Table S2. Primer sequences used in CRISPR/Cas9 genome redaction

Primer pair	Sequences
gRNA#2-sen	5' cttcGTCAGCGACTTGCGGCCCGC 3'
gRNA#2-as	5' aaacGCGGGCCGCAAGTCGCTGAC 3'
gRNA#5s	5' cttcGTCGCTCTGCACATCGAGGA 3'
gRNA#5as	5' aaacTCCTCGATGTGCAGAGCGAC 3'
pr-1	5’ TCCTTCGATAGTTATGACTACCA 3’
pr-2	5' GAAGGCTTTGCTGTGCTGCAGAA 3'

Table S3. Oligos sequences used in RNA interference

Primer pair	Sequences
SuHw_Ri-Fw	5’taatacgactcactatagATAGCGACAGAACTCTAGATGCT 3’
SuHw_Ri-Rev	5’taatacgactcactatagATGAGTGCCTCCAAGGAGGGC 3’
CP190_Ri-Fw	5’ taatacgactcactatagATGGGTGAAGTCAAGTCC 3’
CP190_ Ri-Rev	5’ taatacgactcactatagAGCGAATTCCTTAACCTCTT 3’
Mod_Ri-Fw	5’ taatacgactcactatagATGGCGGACGACGAGCAATTC 3’
Mod_Ri-Rev	5’ taatacgactcactatagCCTCCTCGAGCGCCGG 3’
CP60_Ri-Fw	5’ taatacgactcactatagATGGCAATCCAACTGGAC 3’
CP60_Ri-Rev	5’ taatacgactcactatagCTGAATGGTGGTGCGCGAGG 3’

Table S4. Primer sequences used in ChIP-qPCR analysis

Primer pair	Sequences
62D-Fw<br>62D-Rev	5’ TTTGGGCTTGGTGAGAACAG 3’<br>5’ TGATACCAGGCGAACAGAAATC 3’
50A-Fw<br>50A-Rev	5’ ATACAAAGTGGTTTCAGCCAAGAAG 3’<br>5’ TTGATAAATAGTCCAGCACGCATAC 3’
87E-Fw<br>87E-Rev	5’ GGATGTTACATTGAGAGTGCTTAGG 3’<br>5’ TTTGCGTTTCGGCTGCTGTC 3’
gypsy-Fw<br>gypsy-Rev	5’ TTCTCTAAAAAGTATGCAGCACTT 3’<br>5’ CACGTAATAAGTGTGCGTTGA 3’
1A2-Fw<br>1A2-Rev	5’ ACCACACATCAGTCATCGTGT 3’<br>5’ CTTCGTCTACCGTTGTGC 3’
Ras-Fw<br>Ras-Rev	5’ GAGGGATTCCTGCTCGTCTTCG 3’<br>5’ GTCGCACTTGTTACCCACCATC 3’
Fab3_cts_d<br>Fab3_cts_r	5' TAAAGGCCAATGCACAAAGGCGAC 3’<br>5' ACGCTTCAGCGAACGGAATACAGA 3’
Fab4_cts_d<br>Fab4_cts_r	5’ CAATTTGCCAATATTTCGCAGTCCCT 3’<br>5’ CCCTGGCGGGCATATGAGAAA 3’
MCP_cts_d<br>MCP_cts_r	5’AAAGTCGGGTCTGCAAATAAGG 3’<br>5’ GCATAAGCTGCAAAAGAAAAACAA 3’
Fab6_cts_d<br>Fab6_cts_r	5’ AGCTAAACCCGATTTGCTTTGCCG 3’<br>5’ CTGCCCAGTGGGAGATACAAAGAT 3’
Fab8_cts_d<br>Fab8_cts_r	5’ TGTTGGTGAGCAAGCGAAGA 3’<br>5’ CGAACATTTTTTACGCGACATGT 3’
F7-RTd<br>F7-RTr	5’ TAAGCCAACTGGTTTCCAACTCT 3’<br>5’ TTGCCCAGGGTAAGTAACGGTAT 3’
cg3281_fw<br>cg3282_rev	5’ TGCCCGATAAAATCACAGCTGT 3’<br>5’ CAAATACATCTATCAAAGCGTCTTA 3’
aur-Fw<br>aur-Rev	5’ CTAACACTACAAAGTCACGATATTC 3’<br>5’ CAATGAAAGCCGATATTGGGTAA 3’
janA-Fw<br>janA-Rev	5’ TCGTTCACCTTATGTATCGATAC3’<br>5’ GTTGTGAACGCCAGAGCTGT 3’
Ubx-1<br>Ubx-2	5' CGCCAAGTGCAGTGCAACAG 3'<br>5' CGACAGAGCAAGACAGCAGCA 3'

bxd-1<br>bxd-2	5' GCTGCTCTCTCGCTCTAGCAA 3'<br>5' GAGAGCGATCCGAGCGAGAA 3'
AbdA-pr 1<br>AbdA-pr 2	5' GCCATGCGAGCGTTCTTATGG 3'<br>5' GGCTCCAATAGTTTCCATTCTCAC 3'
iab4-1<br>iab4-2	5' CCTAACCGGTGTGCGAGTAGA 3'<br>5' CCAACCACAAATACACT AGCA 3'
AbdB-1<br>AbdB-2	5' CAGGCGAGAAAAGTGCAGCAG 3'<br>5' CCGCTGATTCATCGGTGGCAA 3'

Table S5. Primer sequences used in amplification of fragments for EMSA

Primer pair	Sequences
EMSA-62D_Fw<br>EMSA-62D_Rev	5’ GCCACGAATGTCCCACGAATC 3’<br>5’ TAGGCCGTTATAAACGGTAGAC 3’
EMSA-50A_Fw<br>EMSA-50A_Rev	5’ TTGATAAATAGTCCAGCACGCATAC 3’<br>5’ CGCTTTCTAGGGACTCGTAATCC 3’
EMSA-87E_Fw<br>EMSA-87E_Rev	5’ GGATGTTACATTGAGAGTGCTTAGG 3’<br>5’ TTTGCGTTTCGGCTGCTGTC 3’
EMSA-66E_Fw<br>EMSA-66E_Rev	5’ CGAACCCTAACCAGTACTAACC 3’<br>5’ GTGAGATCCTTGACCGTGTGG 3’
EMSA-1A2-125_Fw<br>EMSA-1A2-125_Rev	5’ ACCACACATCAGTCATCGTGT 3’<br>5’ CTTCGTCTACCGTTGTGC 3’
EMSA-1A2-250_Fw<br>EMSA-1A2-250_Rev	5’ CTGCAAGCTAGATCCACCTG 3’<br>5’ CAAGAACATTTCCGATATG 3’
EMSA-1A2-450_Fw<br>EMSA-1A2-450_Rev	5’ CGCATCTTCTGGATCATAGGAC 3’<br>5’ CCCTGATTACACAACAAGGTG 3’
Adf1-Fw<br>Adf1-Rev	5’ GAGGCAGCGACTGCGCC 3’<br>5’ GTTGCAACAATCTAATAACAATCCAAG 3’

Supplementary Figures

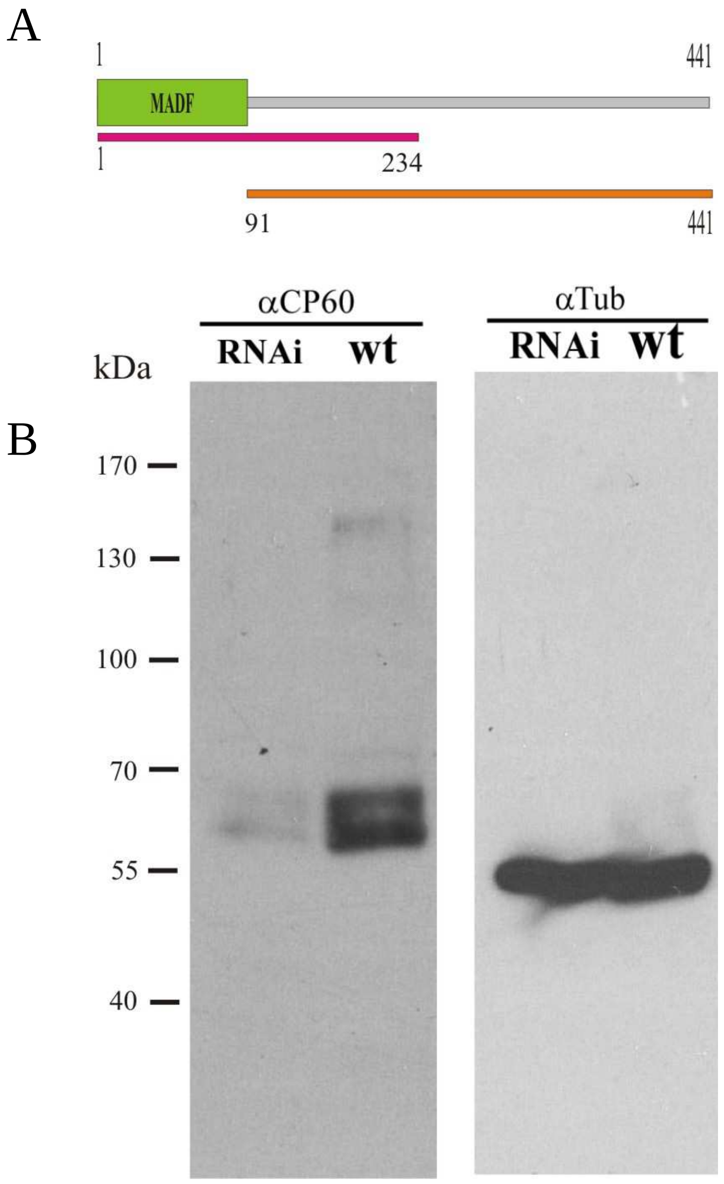

Figure S1. Specificity of CP60 antibodies. (A) Schematic presentation of the CP60 protein. The region of the CP60 protein used to generate antibodies is in orange, while the region used to generate dsRNA is in red. Numbers are the amino acid residues that flank the protein regions. (B) Western blot analysis (8% SDS PAGE) of extracts obtained from S2 cells before (wt) and after (RNAi) treatment with dsRNA to inactivate the *Map60* gene. The membrane was sequentially stained with polyclonal rat antibodies against CP60 (αCP60) and antibodies against Tubulin (αTub) as the loading control. The molecular weight in kDa is marked on the left. No nonspecific signals were detected.

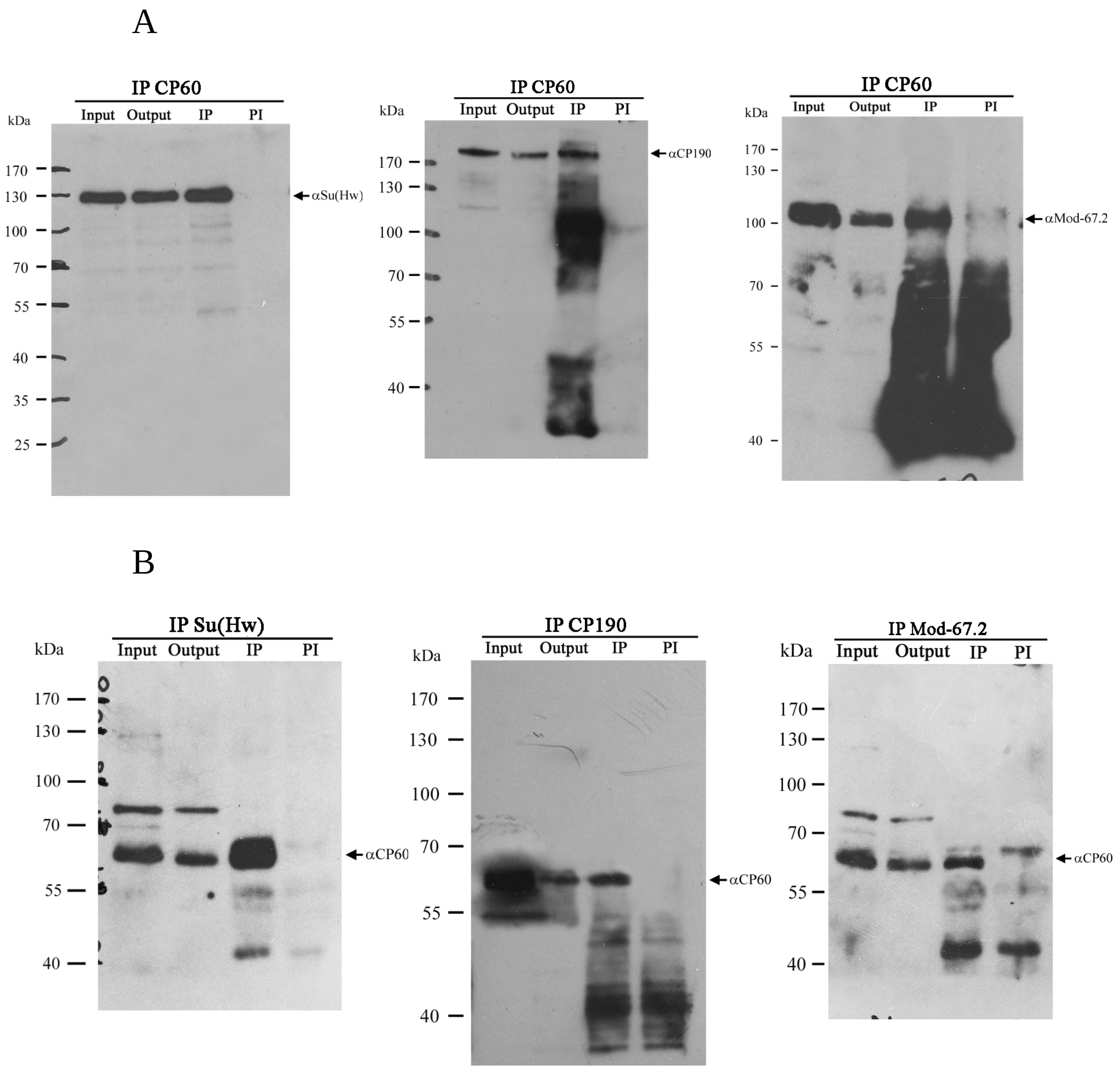

Figure S2. CP60 is a component of the Su(Hw) insulator complex. (A) Nuclear extract from *Drosophila* S2 cells was immunoprecipitated with antibodies against CP60 and immunoprecipitates (IP) were analyzed by Western blotting for Su(Hw), Mod(mdg4)-67.2, and CP190 proteins. (B) Nuclear extract from *Drosophila* S2 cells was immunoprecipitated with antibodies against Su(Hw), Mod(mdg4)-67.2, or CP190, and immunoprecipitates were analyzed by Western blotting for CP60 protein. The molecular weight in kDa is marked on the left. The uncropped images are shown. All designations as in Figure 1.

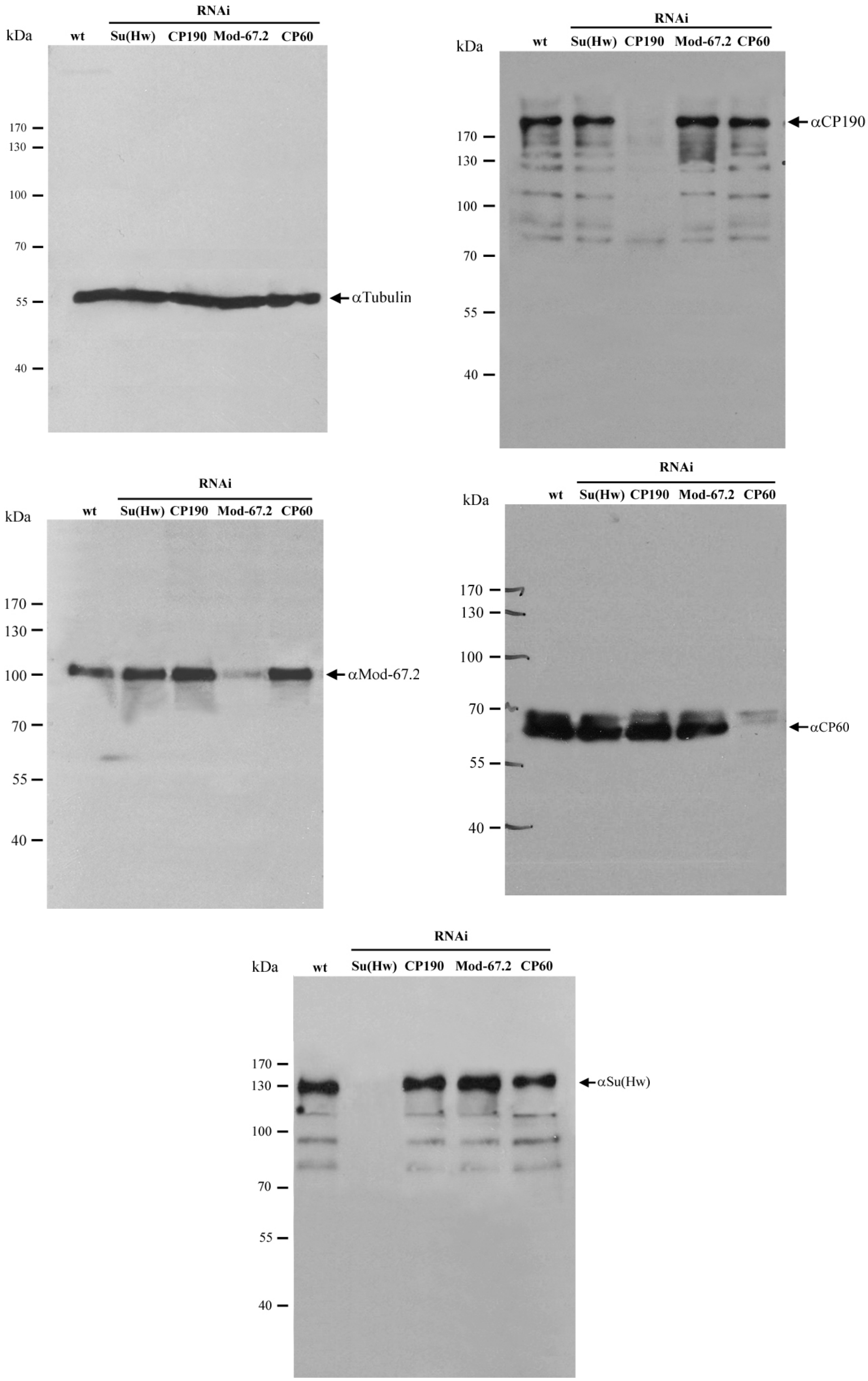

RNAi
kDa
wt
Su(Hw)
CP190
Mod-67.2
CP60
170
130
100
70
55
40
αTubulin
αCP190
αMod-67.2
αCP60
αSu(Hw)

Figure S3. CP190 is responsible for CP60 recruitment to Su(Hw) chromatin sites.

Western blot of RNAi efficiency. wt – S2 cells without treatment; proteins indicated under the line (RNAi) were knockout by RNAi; antibodies for staining are shown on the right; anti-tubulin antibodies (αTub) were used as a loading control. The uncropped images are shown.

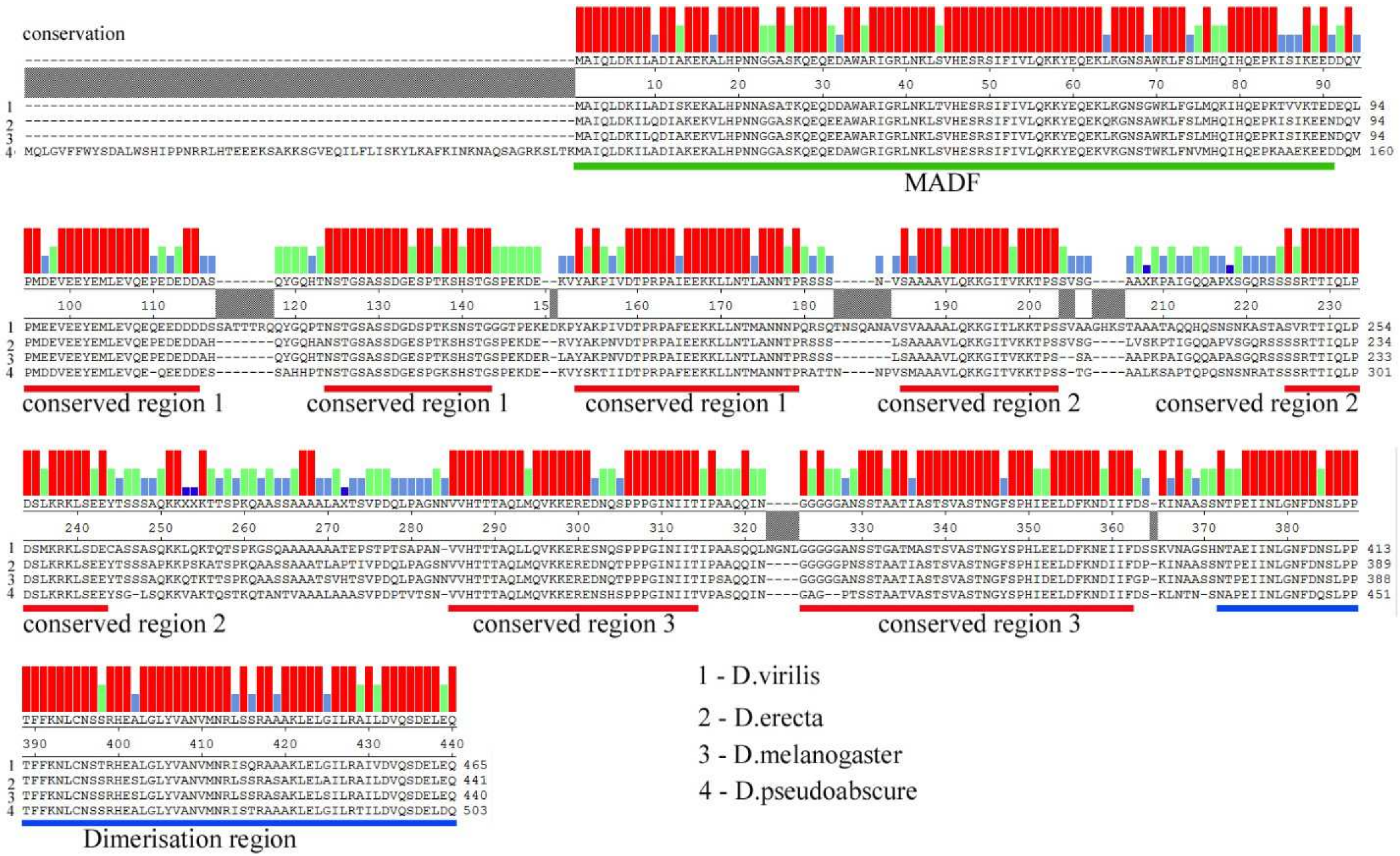

Figure S4. Alignment of CP60 among Drosophilidae. The MADF domain is underlined in green. Conserved regions from 1 to 3 are underlined in red. The fourth conserved dimerization region is underlined in blue.

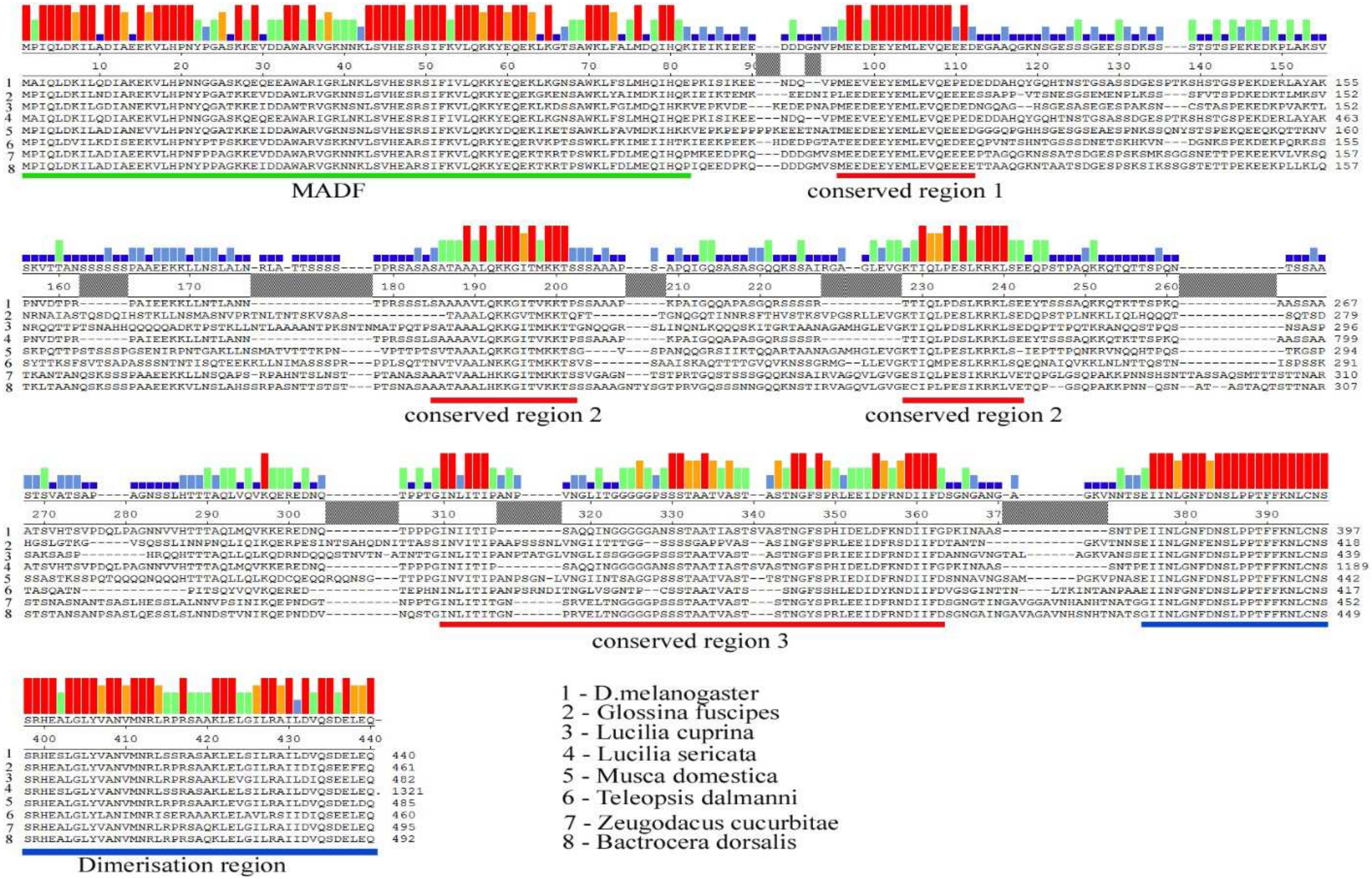

Figure S5. Alignment of the CP60 among Diptera. All designations as in Figure S4.

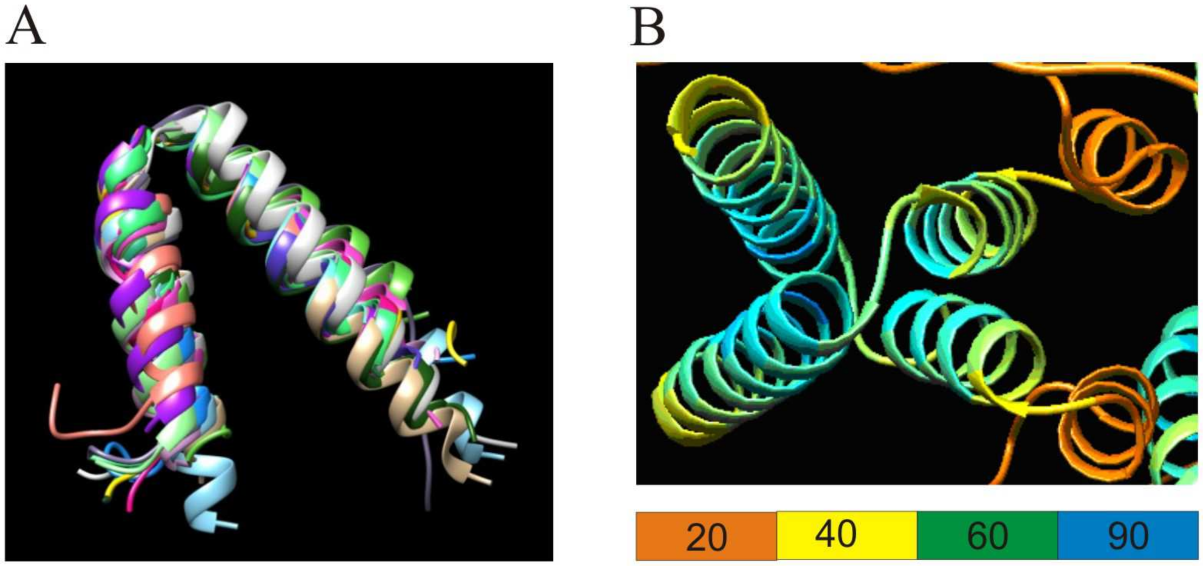

Figure S6. The CP60 C-terminal conserved region has a similar structural organization as the BESS domain. (A) Superposition of the CP60 C-terminal 440–441 aa conserved domain (dark green) structure with different known BESS domain structures. (B) Alphafold prediction of CP60 C-terminal conserved domain (440–441 aa) dimerization. Probability scale in percent is shown at the bottom.

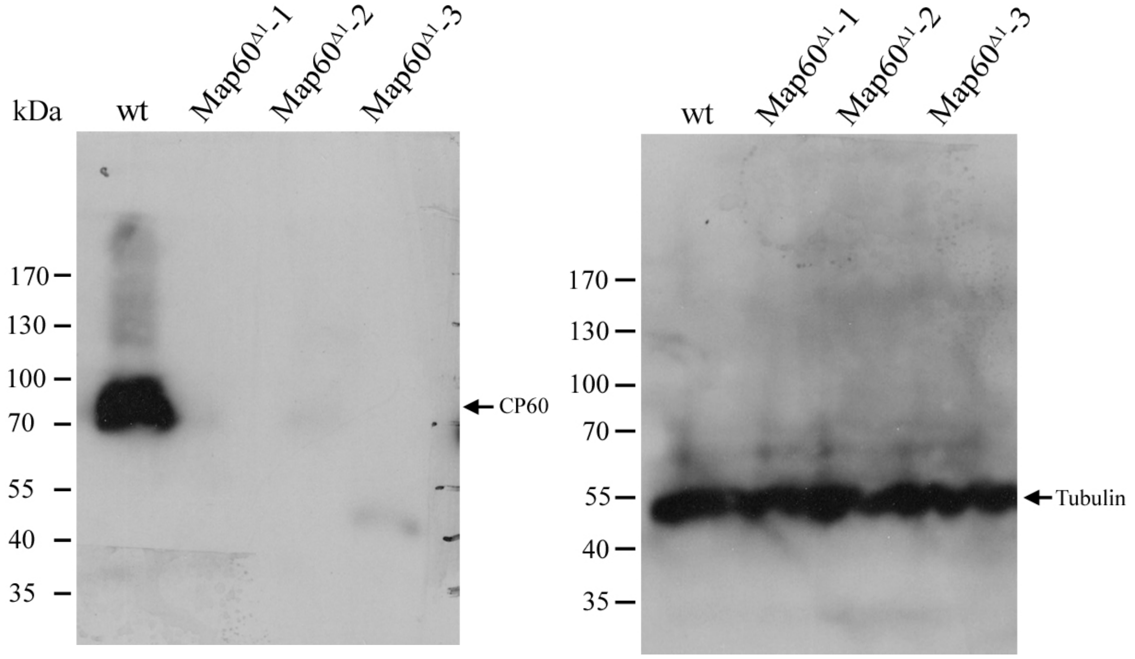

Figure S7. Functional analysis of the *Map60* gene. Western blot analysis (8% SDS PAGE) of protein extracts prepared from adult three-day-old males of the wild-type (wt) line and three lines homozygous for *Map60*$^{\Delta 1}$. The uncropped images are shown. All designations as in Figure 6.

Supplementary Materials

Constructs for yeast two-hybrid assay

Generation of full length Su(Hw), CP190 and Mod-67.2 in pGBT9 and pGAD424 vectors for Y2H assay was described previously [1,2].

To generate **pGBT9 CP60 (full length 1-441)** or **pGAD424 CP60 (full length 1-441)** the coding region of CP60 from pSK CP60 was cloned in vectors pGBT9 or pGAD424 using EcoR1 and BamH1 sites.

To generate **CP60 (1-367)** derivative corresponding fragment was amplified with primers 5'- atagaattcatggcaatccaactggac -3' (upstream, containing EcoRI site) and 5'-ataggatcc gttgatcttcggcccgaag-3' (downstream, containing BamHI site) from pSK CP60. The PCR product was cleaved with BamHI and EcoRI, and the 1100 bp BamHI–EcoRI fragment was cloned into pGBT9 or pGAD424 vector cleaved with the same enzymes.

To generate CP60 (366-441) derivative corresponding fragment was amplified with primers 5'-atagaattcaacgccgcctcctccaatac-3' (upstream, containing EcoRI site) and 5'-ataggatccctgctccagttcgtcg-3' (downstream, containing BamHI site) from pSK CP60. The PCR product was cleaved with BamHI and EcoRI, and the 225 bp BamHI–EcoRI fragment was cloned into pGBT9 or pGAD424 vector cleaved with the same enzymes.

To generate **CP60 (373-441)** derivative corresponding fragment was amplified with primers 5'-atagaattccccgagattatcaacctg -3' (upstream, containing EcoRI site) and 5'-ataggatccctgctccagttcgtcg-3' (downstream, containing BamHI site) from pSK CP60. The PCR product was cleaved with BamHI and EcoRI, and the 205 bp BamHI–EcoRI fragment was cloned into pGBT9 or pGAD424 vector cleaved with the same enzymes.

To generate **CP60 (1-104)** derivative corresponding fragment was amplified with primers 5'- atagaattcatggcaatccaactggac -3' (upstream, containing EcoRI site) and 5'-ataggatcc catttcgtactcctccac-3' (downstream, containing BamHI site) from pSK CP60. The PCR product was cleaved with BamHI and EcoRI, and the 312 bp BamHI–EcoRI fragment was cloned into pGBT9 or pGAD424 vector cleaved with the same enzymes.

To generate **CP60 (1-190)** derivative corresponding fragment was amplified with primers 5'- atagaattcatggcaatccaactggac -3' (upstream, containing EcoRI site) and 5'-ataggatcc actgctgcggcggcgg-3' (downstream, containing BamHI site) from pSK CP60. The PCR product was cleaved with BamHI and EcoRI, and the 570 bp BamHI–EcoRI fragment was cloned into pGBT9 or pGAD424 vector cleaved with the same enzymes.

To generate **CP60 (95-441)** derivative corresponding fragment was amplified with primers 5'-atagaattccccatggaggaggtggag -3' (upstream, containing EcoRI site) and 5'-

ataggatccctgctccagttcgtcg-3' (downstream, containing BamHI site) from pSK CP60. The PCR product was cleaved with BamHI and EcoRI, and the 1040 bp BamHI–EcoRI fragment was cloned into pGBT9 or pGAD424 vector cleaved with the same enzymes.

To generate **CP60 (Δ104-180)** derivative two sets of PCR reaction was made from pSK CP60. First with primers 5'- atagaattcatggcaatccaactggac -3' (upstream, containing EcoRI site) and 5’ –catttcgtactcctccac- 3’ following by digestion with EcoRI enzyme. Second with primers 5’ –agtagctcgctgtccgcc- 3’ and 5'-ataggatccctgctccagttcgtcg-3' (downstream, containing BamHI site) following by digestion with BamHI enzyme. Purified PCR products were simultaneously cloned into pGBT9 or pGAD424 vector cleaved with BamHI and EcoRI.

To generate **CP60 (Δ190-278)** derivative two sets of PCR reaction was made from pSK CP60. First with primers 5'- atagaattcatggcaatccaactggac -3' (upstream, containing EcoRI site) and 5’ – tactgctgcggcggcgg - 3’ following by digestion with EcoRI enzyme. Second with primers 5’ – ctgccggcgggaaacaatgt- 3’ and 5'-ataggatccctgctccagttcgtcg-3' (downstream, containing BamHI site) following by digestion with BamHI enzyme. Purified PCR products were simultaneously cloned into pGBT9 or pGAD424 vector cleaved with BamHI and EcoRI.

To generate **CP60 (Δ285-373)** derivative two sets of PCR reaction was made from pSK CP60. First with primers 5'- atagaattcatggcaatccaactggac -3' (upstream, containing EcoRI site) and 5’ – cacattgtttcccgccgg - 3’ following by digestion with EcoRI enzyme. Second with primers 5’ – cccgagattatcaacctg- 3’ and 5'-ataggatccctgctccagttcgtcg-3' (downstream, containing BamHI site) following by digestion with BamHI enzyme. Purified PCR products were simultaneously cloned into pGBT9 or pGAD424 vector cleaved with BamHI and EcoRI.

Cells lysate preparation and immunoprecipitation experiments

S2 cells grown in SFX medium were collected by centrifugation at 700 x g for 5 min, washed twice with 1×PBS and resuspended in IP-0 buffer (10 mM Tris-HCl, pH 8.0; 10 mM NaCl, 10 mM $MgCl_2$, 1 mM EDTA, 1 mM EGTA, 250 mM sucrose, 1 mM PMSF, 0.2% NP-40, Roche Complete (EDTA-free) Protease Inhibitor Cocktail), incubated for 10 min at 4°C and disrupted by 20 strokes in a Dounce homogenizer on ice. The nuclei were pelleted at 3000 x *g* for 10 min and resuspended in IP-10+ buffer (10 mM Tris-HCl, pH 8.0; 10 mM NaCl, 10 mM $MgCl_2$, 1 mM EDTA, 1 mM EGTA, 10% Glycerol, 0.1% NP-40). An equal volume of IP-850+ (10 mM Tris-HCl, pH 8.0; 850 mM NaCl, 10 mM $MgCl_2$, 1 mM EDTA, 1 mM EGTA, 10% glycerol, 0.1% NP-40) was added, and the nuclei were lysed for 10 min on ice; then two volumes of IP-10+ with 1 U/mL DNase I were added, and incubation was continued for 10 min. at room temperature, with rotation. The lysate was cleared by centrifugation at 16 000 x *g* for 20 min and used in immunoprecipitation assays.

Rabbit antibodies against CP190 (1:500), Su(Hw) (1:1000), Mod(mdg4)-67.2 (1:200) and rat antibodies against CP60 (1:300) were conjugated with either Protein A agarose for rabbit antibodies or Protein G for rat antibodies beads (Pierce, United States). In respective control experiments, rabbit or rat preimmune serum were used. An aliquot of an antibody was mixed with 30 µL of agarose beads equilibrated in IP-150 buffer (10mM HEPES pH=7.5; 150 mM NaCl; 10mM $MgCl_2$; 1mM EDTA; 1mM EGTA; 0.1% NP-40; 10% glycerol) and incubated on a rotator at room temperature for 2 h. The beads were then washed with the same buffer. The nuclear extract (40 mg protein) was adjusted to a volume of 600 µL with IP buffer, mixed with antibody-conjugated beads, and incubated on a rotator overnight at 4°C. Then the beads were washed with two portions of IP-150, one portions of IP-500 (with 500 mM NaCl), and one portion of IP-150, resuspended in SDS-PAGE loading buffer, boiled, and analyzed by Western blotting. Proteins were detected using the ECL Plus Western Blotting substrate (Pierce).

Supplementary references

1. Melnikova, L.; Kostyuchenko, M.; Molodina, V.; Parshikov, A.; Georgiev, P.; Golovnin, A. Interactions between BTB Domain of CP190 and Two Adjacent Regions in Su(Hw) Are Required for the Insulator Complex Formation. *Chromosoma* **2018**, *127*, 59–71, doi:10.1007/s00412-017-0645-6.
2. Melnikova, L.; Kostyuchenko, M.; Molodina, V.; Parshikov, A.; Georgiev, P.; Golovnin, A. Multiple Interactions Are Involved in a Highly Specific Association of the Mod(Mdg4)-67.2 Isoform with the Su(Hw) Sites in Drosophila. *Open Biol* **2017**, *7*, 170150, doi:10.1098/rsob.170150.
